# Periodontitis induces endothelial dysfunction in mice

**DOI:** 10.1038/s41598-021-94418-8

**Published:** 2021-07-22

**Authors:** Maria Parvaneh, Paul K. Witting, Jaqueline Ku, Tala Moradi, Elif Eroglu, Ben Freedman, Greg T. Sutherland, Andrew McCorkindale, Boris Guennewig, Phannaphat Choowong, Kim Bell-Anderson, Gregory Cooney, Shane R. Thomas, Joerg Eberhard

**Affiliations:** 1 Charles Perkins Centre, University of Sydney School of Dentistry, Faculty of Medicine and Health, The University of Sydney, Sydney, NSW Australia; 2 The Charles Perkins Centre, School of Medical Sciences, Faculty of Medicine and Health, The University of Sydney, Sydney, NSW Australia; 3 School of Medical Sciences, The University of New South Wales, Sydney, NSW Australia; 4 Charles Perkins Centre, Heart Research Institute, The University of Sydney, Sydney, NSW Australia; 5 Brain and Mind Centre, School of Medical Sciences, Faculty of Medicine and Health, The University of Sydney, Sydney, NSW Australia

**Keywords:** Arterial stiffening, Experimental models of disease, Periodontitis

## Abstract

The treatment of periodontitis has numerous positive effects on established chronic health conditions, including cardiovascular disease and diabetes. However, ethical considerations do limit the establishment of human trials to investigate whether periodontitis promotes the early stages of chronic conditions. Therefore, the aim of this study was to investigate whether periodontitis induces endothelial dysfunction in hyperlipidemic apolipoprotein E gene-deficient (ApoE^-/-^) mice. Forty-five 8-week-old ApoE^-/-^ mice were challenged by oral lavage with *Porphyromonas gingivalis* and *Streptococcus gordonii* for 4 weeks. A subgroup of animals (n = 15–17/group) was placed in a metabolic chamber immediately before euthanasia at 4 weeks to measure VO_2_/CO_2_ concentrations and voluntary locomotion. In infected and control animals alveolar bone levels were measured by x-ray imaging and endothelial function was determined by measuring endothelial-dependent vasorelaxation of aortic rings. The mRNA expression levels of serum amyloid A and tumor necrosis factor were determined in liver tissues by qRT PCR and protein concentrations in serum by ELISA. Caecal contents were analysed by sequencing to determine changes to the gut microbiota to investigate linkages between microbiome and systemic changes. The results showed that oral lavage of *P. gingivalis* and *S. gordonii* for 4 weeks, initiated periodontitis in ApoE^-/-^ mice, similar to the human situation. The oral inflammation was accompanied by a significant increase in mRNA expression of pro-inflammatory mediators serum amyloid A1 and tumor necrosis factor in the liver. Mice with periodontitis also exhibited impaired endothelial-dependent vasorelaxation responses to acetylcholine. This systemic response was connected to increased energy expenditure, locomotion and respiratory quotient. No differences were detected in caecal microbiota between the infected and control animals. Overall, this is the first report that provide evidence that periodontitis induces endothelial dysfunction in mice. Other systemic responses observed in response to the local reaction need further investigation. The study suggests that early prevention of periodontitis may help limit the early stages of endothelial dysfunction that is linked to atherogenesis in humans.

## Introduction

Worldwide, 743 million people suffer from severe periodontitis making this oral disease the 6th most prevalent disease^1^. Periodontitis is a chronic inflammatory disease caused by the accumulation and enrichment of opportunistic bacteria on dental surfaces including gram-negative anaerobic bacteria such as *Porphyromonas gingivalsis (P. gingivalis), Treponema denticola* and *Tannerella forsythia.* Bacterial products and the host immune response cause a local chronic inflammation and resulting in the loss of tooth supporting tissues. A recent workshop held by the World Heart Federation and the European Federation of Periodontology concluded that there is robust evidence that periodontitis is an independent predictor of coronary artery disease and myocardial infarction in humans^2^ and intervention studies have recently demonstrated that adequate treatment of periodontitis concomitantly decreases surrogate biomarkers and risk indicators for cardiovascular events^3,4^. However, a direct causal link between periodontal disease and factors contributing to cardiovascular disease has not been shown yet, because of a lack of any prospective trial of periodontal intervention for the primary prevention of cardiovascular diseases.

A healthy vascular endothelium is essential for the maintenance of cardiovascular homeostasis and the prevention of atherosclerosis. Endothelial dysfunction is an early pathogenic event of atherosclerosis, essential hypertension, and related cardiovascular disorders^5^. Following these initial events, a combination of inflammatory processes and dysregulated lipid metabolism promotes atherosclerotic plaque formation in the arterial wall^6^. In support of this conclusion, a large-scale observational study has shown that periodontal disease severity was significantly associated with endothelial dysfunction as measured by flow-mediated dilation^7^. This observation was confirmed in subjects without cardiovascular risk using strain-gauge plethysmography to measure forearm blood flow^8^. In addition, clinical trials involving patients with cardiovascular risk have identified improved endothelium-dependent vasorelaxation after intensive periodontitis treatment compared to controls^4,9^.

Although these studies support the concept that extravascular inflammation may contribute to the development of endothelial dysfunction as an early stage of cardiovascular pathologies the feasibility of performing human trials in primary prevention is hampered due to ethical and methodological considerations. Therefore, the current study design investigated whether induction of periodontitis in hyperlipidemic apolipoprotein E gene-deficient (ApoE^-/-^) mice promotes systemic inflammation and endothelial dysfunction, prior to the development of extensive atherosclerotic lesions. In addition, we aimed to quantify the acute-phase response in the liver to show potential pathways between the local and systemic reaction.

## Materials and methods

### Animals

Eighty-one 8-week-old male ApoE gene knockout (ApoE^-/-^) (B6.129P2-Apoetm1Unc/J in a C57BL/6 background) mice with a mean body weight of 25.5 g were purchased from Animal Resources Centre (Canning Vale, WA, Australia). The number of animals for each group was based on previous experiments. Prior to experimentation, the mice were housed for one-week in the animal facilities of the Charles Perkins Centre, The University of Sydney, at 22 ± 2 °C with a 12-h light/dark cycle. All mice were fed with normal mouse chow (Speciality Feeds, WA, Australia) and water was provided ad libitum. The study was carried out in compliance with the ARRIVE guidelines^10^ and all procedures were performed in accordance with institutional guidelines for animal research and were approved by the local government authorities in compliance with international laws and policies (Animal Ethics Committee, Animal Research Authority of the University of Sydney, project number 2017/1145; date of approval: 17 May 2017).

### Preparation of bacterial culture and oral infection

*P. gingivalis* strain ATCC 3327 was grown in a medium containing Brain Heart Infusion (BHI; Thermo Fisher Scientific, Scoresby, VIC, Australia) with 1.5% agar supplemented with, 0.5% w/v Yeast extract (if not otherwise stated all chemicals were ordered from Sigma-Aldrich, St. Louis, MI, United States), 0.05% w/v L-Cysteine, 4% v/v sheep blood (Thermo Fisher Scientific, Scoresby, VIC, Australia), 0.2% w/v of menadione 0.5 mg/mL and 1% w/v of hemin 0.5 mg/mL. Bacteria were incubated at 37 °C for 48 h in an anaerobic chamber (Bugbox, Baker, Sanford, MA, United States) with 10% CO_2_, 5% H_2_ and 85% N_2_. A single colony was isolated from the plates and was grown in a BHI broth supplemented as described above (excluding sheep blood) and incubated under the same conditions. *Streptococcus gordonii* (*S. gordonii*) strain ATCC 10558 was grown on Columbia Blood Agar (Thermo Fisher Scientific, Scoresby, VIC, Australia) supplemented with 5% (v/v) sheep blood and incubated at 37 °C for 24 h in 10% CO_2_. A single colony of the bacteria was isolated from the plate and inoculated in BHI broth media supplemented with 0.3% (w/v) yeast extract and incubated for 24 h at 37 °C. *P. gingivalis* concentration was determined in broth media at late log phase at a wavelength of 600 nm (OD = 1.5) and *S. gordonii* concentration were determined at a wavelength of 600 nm (OD = 1.2), followed by serial dilution with growth media and harvest by centrifugation and resuspension in a ratio of 1:1 (v/v) in 2% (w/v) Carboxyl Methyl Cellulose (CMC; Alpha Chemicals, Wetherill Park, NSW, Australia) in Phosphate Buffered Solution (PBS; Sigma-Aldrich, St. Louis, Missouri, United States). Next ApoE^-/-^ mice (9 weeks of age) were infected with *P. gingivalis/S. gordonii* (0.2 mL of 10^10^–10^11^ CFU/mL each); animals were randomly selected using a random numbers table (n = 45) and infected via oral lavage of 0.4 mL twice a day for 4 weeks. Mice assigned to the vehicle (control group; n = 36) received CMC in PBS in the absence of the bacterial load. For oral lavage the bacterial solution or CMC vehicle was sequentially applied in increments of approximately 50 µL over a period of 1 min into the oral cavity of the mouse using a 1 mL laboratory pipette (Eppendorf, Hamburg, Germany).

### Respirometry

Oxygen consumption rate (VO_2_) and carbon dioxide production rate (VCO_2_) were measured under a consistent environmental temperature (22 °C) using an indirect calorimetry system (Promethion, Sable System International, North Las Vegas, NV, United States). For mice, studies were commenced after 4 h of acclimation to the home-style metabolic chamber using an air flow of 2 L/min. VO_2_ and VCO_2_ of individual mice was measured at 5-min intervals over a 24 h period. Mice had ad libitum access to food and water. The respiratory quotient (RQ) was calculated by dividing VCO_2_ by VO_2_. Physical activity was calculated from voluntary locomotion in individual mice in the metabolic cages measured by X, Y and Z beam breaks.

### Animal sacrifice and tissue harvesting

After 4 weeks, mice were anaesthetised using inhaled isoflurane (3% v/v), sacrificed and blood was taken via cardiac puncture and subsequently serum was separated. The heart and aorta were perfused with ice-cold PBS until a pale colour of the liver was observed. The thoracic aorta close to the abdomen was carefully isolated for the assessment of vascular function while fresh. The liver was harvested and snap-frozen and the alveolar bones were dissected and cleaned. Next, caecal contents were collected and snap-frozen for the analysis of microbiota composition. Notably, personnel undertaking the following laboratory procedures were blinded to the group assignment of the animals.

### Determination of alveolar bone level

Maxillae were excised, fixed in 4% w/v PFA-polymer (Abcam, Melbourne, VIC, Australia) in PBS for 48 h at 4 °C and stored in PBS at 4 °C until further processing. To determine alveolar bone level dissected maxillae were captured by dual-energy x-ray absorptiometry imaging at 10× magnification (Ultrafocus DXA, Hologic, Marlborough, MA, United States) and the distance between the cemento-enamel junction (CEJ) and alveolar bone crest (ABC) was measured mesial and distal at the right and left second molar using the Faxitron Bioptics software (Hologic, Marlborough, MA, United States). After calibration of the system for the measurement of distances the CEJ was indicated on the mesial and distal surface projection of the tooth and connected with a line drawn in silico. Next, perpendicular lines were drawn through the CEJ to the alveolar bone crest and the distance was automatically measured by the computer-assisted system. All measurements were done by one blinded investigator.

### Vascular function assay

Mouse thoracic aorta were excised and placed in ice-cold Krebs–Henseleit buffer (NaCl, 118.4 mM; KCl, 4.7 mM; CaCl_2_, 2.5 mM; MgSO_4_, 1.2 mM; KH_2_PO_4_, 1.2 mM; NaHCO_3_, 25.0 mM; EDTA, 0.023 mM/L; and glucose, 11.1 mM/L, pH 7.4). Freshly isolated thoracic aortae were cleaned of any perivascular fat and connective tissue using a dissection microscope then cut into 2–3 mm long aortic ring segments. The aortic rings were then mounted in individual organ chambers of a multi-wire myograph system (MultiMyograph 610 M; Danish Myo Technology, Denmark) containing carbogen-bubbled (95% v/v O2, 5% v/v CO_2_) Krebs–Henseleit buffer and maintained at 37 °C. Isometric tension was monitored with a force transducer connected to a data acquisition system. Following a 30 min equilibration period, the resting tension of each vessel preparation was raised to 9.8 mN. The rings were maintained at a baseline tension of 9.8 mN for a further 30 min, followed by the addition of high potassium physiological saline solution (KPSS) containing 120 mM of KCl to induce maximal vessel contraction. The aortic rings were washed with Krebs–Henseleit buffer and allowed to relax to baseline. The contractile responses to KPSS were repeated twice. The aortic rings were then maintained at baseline tension for 30 min and incubated with indomethacin (10 µM; to inhibit the generation of vasoactive prostanoids by cyclooxygenase). Aortic rings were then pre-contracted with U46619 (Sigma-Aldrich, St. Louis, MI, United States) (1–100 nM; ~ 70% of the maximal contraction to KPSS). Once the contractile responses were stable, endothelial-dependent vasorelaxation responses were assessed by performing cumulative concentration–response curves to acetylcholine (1–3000 nM; Sigma-Aldrich, St. Louis, MI, United States). Endothelium-independent vasorelaxation responses were recorded in response to sodium nitroprusside (SNP; 0.1–300 nM). All responses were recorded using the PowerLab data acquisition system (AD Instruments, Bella Vista, NSW, Australia). Vasorelaxation responses were expressed as the percent relaxation of the submaximal contraction in response to U46619.

### qRT PCR of Saa and Tnf gene expression in liver tissue

Total tissue RNA was isolated from approximately 30 mg of liver tissue using the RNeasy mini kit (Qiagen, Hilden, Germany) based on the manufacturer’s protocol. High quality RNA (absorbance ratios at A_260_nm/A_280_nm and A_260_nm/A_230_nm were consistently between 1.8 and 2) was reverse transcribed to cDNA using i-script cDNA synthesis kit (Bio-Rad Laboratories, Gladesville, NSW, Australia) and standard protocols. Quantitative real-time PCR was performed using SYBR Green (Bioline, Memphis, TE, USA) and a light cycler (Light Cycler 480, Roche, Hawthorn, VIC, Australia) with initial denaturation at 95 °C for 2 min, followed by at 95 °C for 5 s, 60 °C for 10 s for 35 cycles. The data were calculated as fold changes mRNA expression relative to the housekeeping gene *beta-actin* (accession no. NM_001313923.1) forward 5′-CACCATTGGCAATGAGCGGTTC-3′, reverse 5′-AGGTCTTTGCGGATGTCCACGT-3′. The examined oligonucleotide primer pairs were as follows: *Tnf* (accession no. XM021149735.1) forward 5′-ATGGCCTCCCTCTCATCAGT-3′, reverse 5′-GTTTGCTACGACGTGGGCTA-3′; *Saa1* (accession no. M13521) forward 5′-ATGAAGGAAGCTAACTGGAAAAACTC-3′, reverse 5′-TCCTCCTCAAGCAGTTACTA-CTGCAA-3′; *Saa2* (accession no. NM_001379269) forward 5′-ATGAAGGAAG-CTGGCTGGAAAGATGG-3′, reverse 5′-TCCTCCTCAAGCAGTTACTACTGCTC-3′; *Saa3* (Accession number NM_011315.3) forward 5′-AAGTTCACGGGACATGGAGC-3′, reverse 5′-GTAGTTGCTCCTCTTCTCGGG-3′. The apolipoproteins serum amyloid A1 (SAA1) and serum amyloid A2 (SAA2) are closely related acute phase proteins. Their serum concentrations can increase by as much as a 1000-fold after induction of hepatic production in response to a range of pro-inflammatory stimuli^11^. Serum amyloid A3 (SAA3) is a pseudogene in humans and no *SAA3* mRNA or SAA3 protein has been identified in humans. Saa3 is commonly secreted by cells such as macrophages and adipocyte in species such as mice and has been detected in blood after high dose LPS injection^12^.

### Enzyme-linked immunosorbent assay (ELISA) of serum cytokines

Standard commercial ELISA kits were used for the quantification of Tnf (BMS607-3, Thermofisher, Scoresby, VIC, Australia) and Saa (ab157723, Abcam, Cambridge, MA, USA) in serum. The concentration levels of cytokines were determined by standard curves for each cytokine using a plate reader at an absorbance of 450 nm (TECAN, Port Melbourne, VIC, Australia) and reported as pg/mL for Tnf and ng/mL for Saa.

### Gut microbiome and sequence analysis

Cecal content was obtained by aseptically extruding material from the cecum. Total DNA was extracted from caecal samples using the DNeasy PowerSoilTM Kit (Qiagen, Hilden, Germany) according to the manufacturer’s instructions, with the addition of a 10-min incubation step at room temperature before the final centrifugation and elution step to increase DNA recovery. All samples were co-extracted with blanks to monitor for contamination. The microbial community was profiled using the 16S rRNA V3–V6 region (nucleotide position 338-1046). Sequencing with Illumina MiSeq was performed at the Ramaciotti Centre for Genomics (University of New South Wales, Sydney, NSW, Australia). Paired-end reads were aligned using Pandaseq. Aligned reads were partitioned into operational taxonomic units (OTUs) by minimum entropy decomposition^13^. OTUs that contained < 0.01% of total reads were filtered out. Taxonomy was assigned to OTUs against the 99% GreenGenes v.13.5 database with UCLUST^14^. Representative sequences were aligned (MAFFT) and used to build a phylogenetic tree (Fasttree). The sequence analysis output of 16S data from QIIME2 was imported into R (version 4.0.0) for downstream statistical analysis^15^. Samples were removed if they had a sequence depth below the sequences cut-off (mean—2*SD), and OTUs > 0. Boxplots of the most relative abundance oral microbiome species in each group were generated to visualize differences in the overall relative abundance of the most prevalent species between groups (R version 4.0.0, Phyloseq version 1.32.0)^16^. Prior to the analysis, samples were normalized using cumulative sum scaling (CSS) with MetagenomeSeq R package to remove biases from sequence depth variation between samples^17^. Relative abundance was converted into percentages, and species with low relative abundance were grouped for ease of visualisation. OTUs with similar species identification were pooled together for relative abundance analysis.

For Beta diversity Unweighted UniFrac distances were calculated by GUniFrac R package (version 1.1)^18^ and displayed as Principal Coordinates Analysis (PCoA) graph based on the calculated unweighted UniFrac distances to visualize dissimilarities and grouping of data. Permutational multivariate ANOVA (PERMANOVA) was used to assess statistically significant differences in the phylogenetic makeup of the data, using vegan R package (version 2.5.6)^19^.

### Statistical analysis

The primary outcome was endothelial-dependent vasorelaxation response in rings of the isolated thoracic aortae. Data were calculated as means ± standard deviation (SD) or means ± standard error (SE) where appropriate. Treatment groups were compared using a t-test for independent variables for data with normal distribution or a Mann–Whitney U Test for data with a non-parametric distribution (defined by the Shapiro–Wilk normality test). For all statistical analyses STATA 15.0 was used (StataCorp LLC, College Station, Texas, USA). The concentration-dependent vasodilation curves were analysed using 2-way ANOVA for repeated measurements.

## Results

### Polymicrobial lavage with P. gingivalis and S. gordonii causes alveolar bone loss

**Figure 1:**
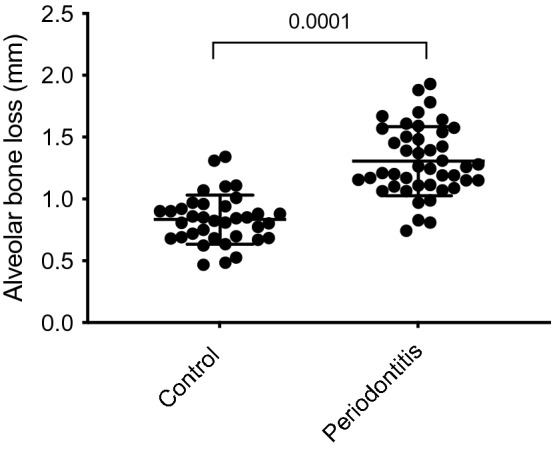
ApoE^-/-^ mice were orally infected with 0.4 mL *P. gingivalis* and *S. gordonii* (10^10^–10^11^ CFU/ml) twice a day for 4 weeks while control group received 0.4 mL of 2% CMC. Alveolar bone level was analysed at the right and left second molar of the upper jaw. The distance between the CEJ and the ABC are presented in mm (control group n = 36; periodontitis group n = 45).

Mice receiving oral lavage with a mixture of *P. gingivalis* and *S. gordonii* over a period of 4 weeks showed a significantly (*P* = 0.0001) decreased alveolar bone crest height (1.33 ± 0.04 mm) compared to the control group (0.83 ± 0.03 mm); this data provided evidence for the successful initiation of an inflammatory response in periodontal tissues of mice with a clinical outcome similar to humans suffering from periodontitis (Fig. 1).

### Systemic SAA and TNF

**Figure 2:**
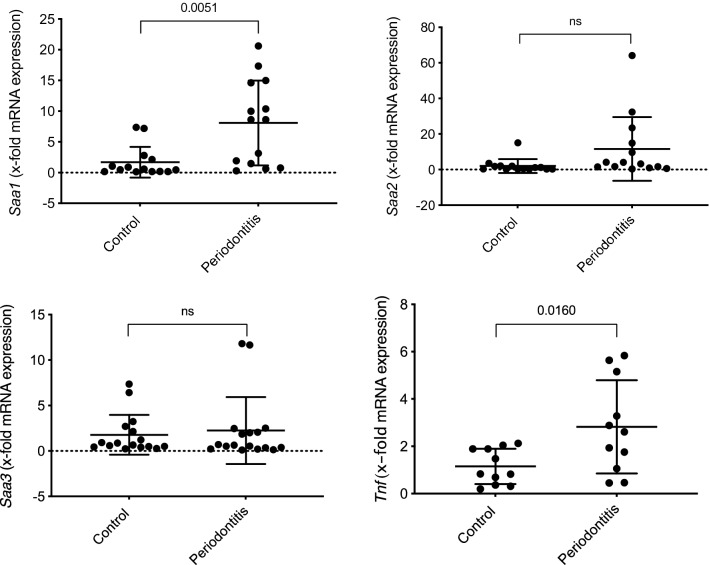
Assessment of mRNA expression levels in liver tissues presented as mean differences (MD) between the infected and control groups (n = 15 mice per group): *Saa1* (MD 6.40 ± 1.97, 95%CI 2.37; 10.43; *P* = 0.0031), *Saa2* (MD 9.58 ± 4.90; 95%CI − 0.50; 19.66: *P* = 0.0615), *Saa3* (MD 0.47 ± 1.06; 95%CI − 1.70; 2.63; *P* = 0.6618) and *Tnf* (MD 1.68 ± 0.64; 95%CI 0.35; 3.00; *P* = 0.0160).

**Figure 3:**
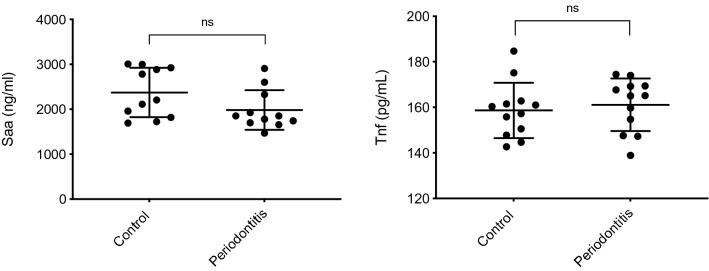
Serum concentrations of Saa and Tnf expressed as MD between the infected and control group with n = 12: Saa (MD − 390.2 ± 212.0, 95%CI − 832.4; 52.0; *P* = 0.32) and Tnf (MD 2.43 ± 4.84; 95%CI − 7.61; 12.47; *P* = 0.62).

In the liver increased mRNA expression of *Saa1* (fold expression mean difference (MD) 6.40 ± 1.96; 95%CI 2.37; 10.43; *P* = 0.0051) and *Tnf* (x-fold expression MD 1.68 ± 0.64; 95%CI 0.35; 3.00; *P* = 0.0160) was observed in the infected compared to the control mice (Fig. 2). No significant difference between the infected and control animals was observed for the mRNA expression of *Saa2* (x-fold expression MD 9.58 ± 4.90; 95%CI − 0.50; 19.66; *P* = 0.0615) and *Saa3* (x-fold expression MD 0.47 ± 1.06; 95%CI − 1.70; 2.63; *P* = 0.6618) (Fig. 2). In serum no statistical significance differences in the levels of Saa (MD − 390.20 ± 212.0 ng/mL; 95%CI − 832.4; 52.00; *P* = 0.3226) and Tnf (MD 2.43 ± 4.84 pg/mL; 95%CI − 7.61; 12.47; *P* = 0.6208) were detected between treatment groups (Fig. 3).

### Periodontitis elicits endothelial dysfunction

**Figure 4:**
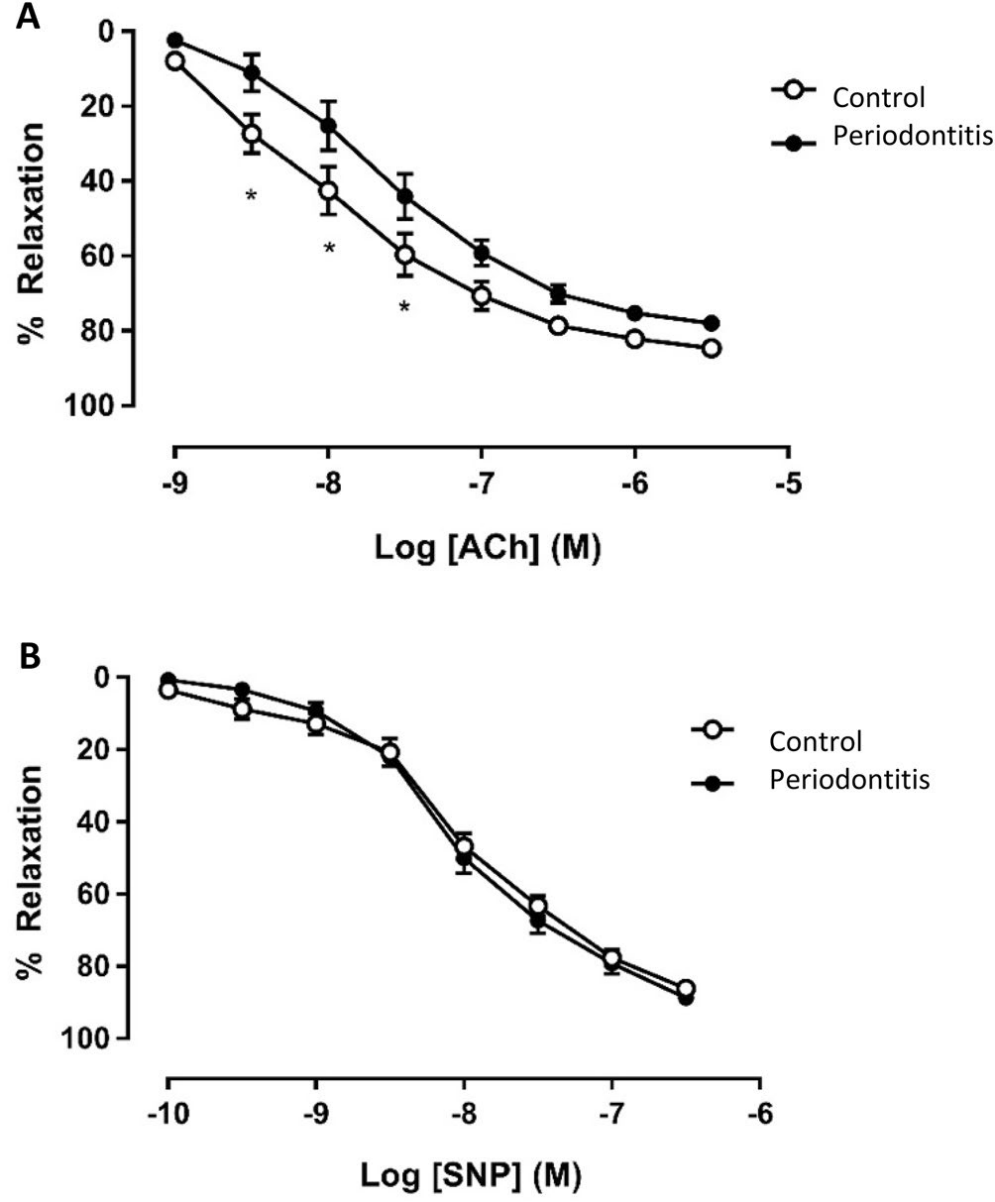
After 4 weeks of oral lavage with *P. gingivalis* and *S. gordonii* (10^10^–10^11^ CFU/ml), endothelial-dependent and -independent relaxation responses of pre-constricted aortic rings were monitored in response to acetylcholine (Ach) or sodium nitroprusside (SNP), respectively and, presented as mean ± SE; A: concentration response curve to Acetylcholine (Ach); B: concentration response curve to sodium nitroprusside (SNP) (n = 8 in each group). *Different to the control; *P* < 0.05 (2-Way ANOVA).

Mice induced to develop periodontitis showed significantly impaired endothelium-dependent relaxation response of aortic rings to acetylcholine at concentrations of 3.16 to 31.62 nM Ach compared to the control group (*P* < 0.05). By contrast, no significant difference was observed in the relaxation of isolated mouse aortic rings in response to the NO-donor sodium nitroprusside indicating NO-independent vasodilation was preserved (Fig. 4).

### Respirometry showed reduced activity in periodontitis-infected mice

**Figure 5:**
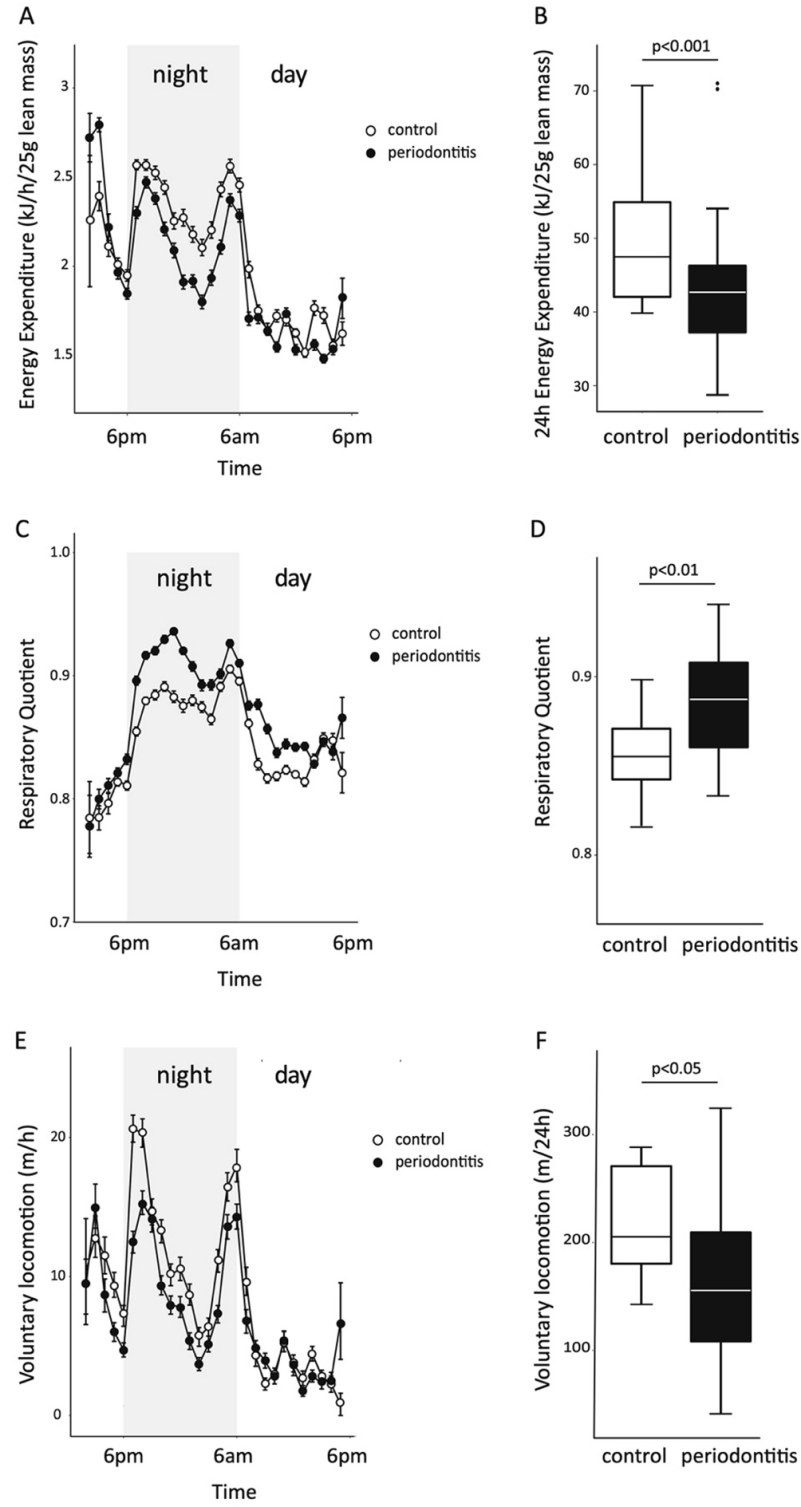
At the end of week 4 mice were placed in a Promethion metabolic chamber in the late afternoon and measurements commenced after 4 h of acclimation and continued for 24 h. Data are depicted as mean ± SE, (n = 15–17 in each group). Significant differences were determined (as indicated in the figure) between the periodontitis and control groups for the parameter energy expenditure (*P* < 0.01), respiratory quotient (*P* < 0.01) and voluntary locomotion (*P* < 0.05).

At the end of the 4 weeks experimental phase animals were kept for 24 h in a Promethion metabolic chamber and data compiled in Fig. 5. Significant differences were detected between the periodontitis and control group over 24 h for total energy expenditure corrected for lean mass due to differences in night-time energy expenditure (periodontitis = 43.6 kJ/24 h/25 g lean mass vs. control = 49.8 kJ/24 h/25 g lean mass; *P* < 0.001) (Fig. 5A,B), respiratory quotient (periodontitis = 0.885 vs. control = 0.858; *P* < 0.01) (Fig. 5C,D) and voluntary locomotion (periodontitis = 171 m/24 h vs. control = 220 m/24 h; *P* < 0.05) (Fig. 5E,F).

### Gut microbiota

**Figure 6:**
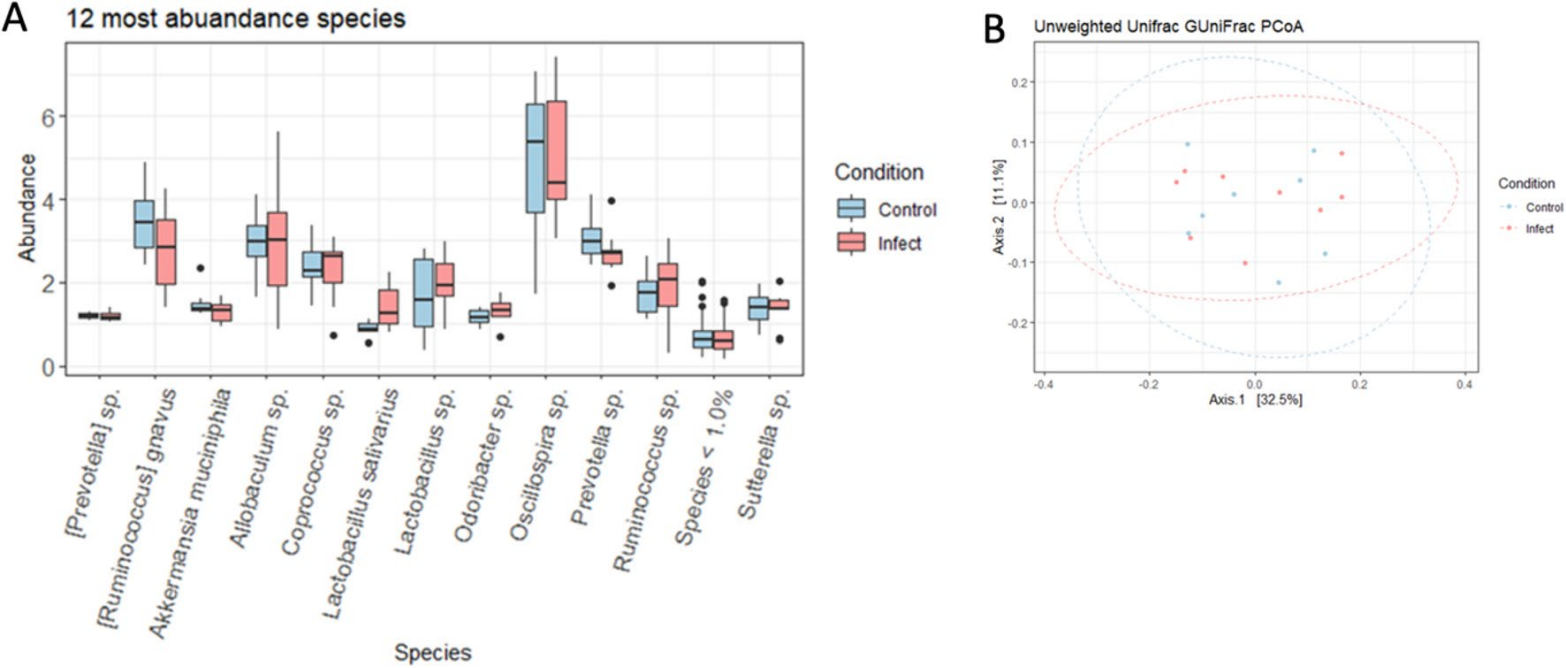
(**A**) Relative abundance in caecal samples of infected and control animals. Prior to overall relative abundance analysis, data undergoes CSS normalisation and abundances were converted into percentages before sorting at species level. OTUs with similar species identification were pooled together for relative abundance analysis. Species with relative abundance < 1.0% were grouped together. (**B**) Beta diversity using Unweighted UniFrac distance (PERMANOVA of LMM condition *P* = 0.90, batch effect *P* < 0.05). Prior to beta diversity analysis, OTUs < 0.01% were judged to be insignificant not included in the overall analysis.

The most abundant species in caecal samples of control and periodontitis infected mice were *Oscillospira sp., Ruminococcus gnavus, and Lactobacillus sp*. (Fig. 6A). The (beta) sample diversity between control and periodontitis infected mice did not show a significant impact of the treatment on the shared microbiome diversity (*P* = 0.90) (Fig. 6B), suggesting that any systemic changes noted above were independent of altered microbiome.

## Discussion

In this study we evaluated the association between the early changes in atherosclerosis with periodontal disease. We induced periodontitis in ApoE^-/-^ mice by oral lavage with *P. gingivalis* and *S. gordonii* and provide evidence that periodontitis resulted in endothelial dysfunction within a period of 4 weeks. The extravascular periodontal inflammation was accompanied by increased mRNA expression of *Saa1* and *Tnf* in liver tissues and whole-body metabolic changes but did not lead to changes of the gut microbiota.

To the best of our knowledge, this is the first report that demonstrates changes of endothelium-dependent vasodilation following *P. gingivalis* and *S. gordonii* oral infection and the concordant development of periodontitis as indicated by alveolar bone loss in infected mice. This study used male ApoE^-/-^ mice, because of the more severe vascular pathology developed in males compared to female mice^20^. The combination of bacteria was selected because of beneficial relationships that have been described between oral streptococci and *P. gingivalis* that support adherence and biofilm development^21^. The impaired endothelial-dependent vasorelaxation of the aorta from infected mice is indicative of the impairment of NO bioactivity and onset of endothelial dysfunction^6^ which is considered an important early event in atherosclerosis^5^. In agreement, Campi et al. demonstrated endothelial dysfunction in rats after 7 days of ligature-induced periodontitis and did not use human bacterial pathogens, which limits the direct translation of these data to the human situation^22^. However, not all available data is supportive. For example, Pereira et al. infected 18 weeks old adult C57 and ApoE^-/-^ mice with the periodontal pathogen *P. gingivalis* over a period of 12 weeks and did not observe changes in endothelium-dependent or -independent vasodilation^23^. One explanation for these opposing results is the use of adult mice (30 weeks at the time of culling) which is close to the middle ages of 40–60 weeks when senescence begins in mice^24^ and significant vascular pathologies may have already established. In contrast, animals were 13 weeks old at the end of the current experiments and therefore, were within the early stages of vascular pathogenesis.

The relationship between periodontal infection and endothelial dysfunction has been investigated in epidemiological studies and clinical trials in humans and consistently show a positive association between periodontitis and endothelial dysfunction^7,8^ and the improvement of endothelium-dependent vasorelaxation after intensive periodontitis treatment compared to control groups^4,9^. Despite the fact that a prospective study in humans investigating whether periodontitis elicit early changes of atherosclerosis is not feasible, the current research in mice is novel and adds to the cumulative evidence that periodontitis is an independent risk factor for cardiovascular conditions and the treatment of periodontitis is likely to reduce the risk for developing cardiovascular disease in otherwise healthy people. This conclusion aligns with human studies that showed that a mild inflammatory reaction in the oral cavity enhance monocyte adherence to endothelial cells and increased foam cell formation after oxLDL uptake, which are also early events related to atherosclerosis^25^.

The local periodontal inflammatory response was accompanied by a systemic response of the liver by increased mRNA expression of acute-phase response proteins Saa and Tnf. However, we were not able to measure an increase of these proteins in serum in the infected animals. Circulating SAA is produced principally by the liver, and levels rise 1000-fold in the acute-phase responses^26^. Elevated levels of SAA are evident in chronic diseases, including diabetes and atherosclerosis^27,28^, and predict cardiovascular disease risk^5^. However, whether SAA is merely an inflammation marker or mediates atherogenesis is debated^29^. Similarly, TNF is an acute phase protein and its increased gene expression in the liver of rats after experimental periodontitis has been demonstrated^30^. The systemic response in the liver highlights a potential pathway for how extravascular periodontitis may affect endothelial function, although, more detailed analyses are necessary to link these pro-inflammatory mediators with the endothelial dysfunction demonstrated here.

Both animal and human studies indicated that *P. gingivalis* may influence the gut microbiota causing dysbiosis^31^ and potential changes of the gut microbiome may be associated with gut microbiota-derived metabolites that drive a systemic response that actively engages in the modulation of peripheral immune responses^32^. Based on the overall relative abundances, in the current study there was a lack of clear difference in the gut microbiota between the periodontitis and control groups. This observation supports the concept that despite the high number of bacteria used for the oral lavage procedures, the observed systemic effects are a consequence of the extravascular oral inflammation, rather than altered gut microbiota.

In this study we also investigated any effect of the periodontal infection on energy expenditure, respiratory quotient and physical activity. Inflammation increases the need of immune cells for rapid generation of cellular energy. To meet this need, immune cells shift to aerobic glycolysis for energy production^33^. The respiratory quotient was significantly increased over 24 h and during the night phase in mice with periodontitis. This may highlight the state of inflammation in periodontitis, which is selective for a state of carbohydrate oxidation rather than fat to meet energy demands. Usually, the respiratory quotient is reflective of the macronutrient composition of the diet and both groups consumed chow which is ~ 65% energy as carbohydrate and low in fat, however the infected group tended to eat more during the night-time, which may explain a greater utilisation of carbohydrate as a reflection of more carbohydrate digestion and absorption. Alternatively, the increased utilisation of carbohydrate over fat may reflect the body composition of these animals which was found to be leaner and had less fat mass (both as absolute weight and as percentage of body mass) than controls (data not shown). Twenty-four hours energy expenditure adjusted for lean mass was significantly reduced in the infected group, which could be explained by the reduction in physical activity that was significantly reduced especially during the night. Interestingly, mice challenged with the obligate intracellular Gram-negative bacterium *Orientia tsutsugamushi* showed changes of lipid and carbohydrate metabolism, as well as significantly altered markers of stress and food intake, pointing towards the generalisation of the observed whole body effects after bacterial challenge in the present experiments^34^. Understanding how periodontitis as a low-grade chronic inflammation modulates body energy metabolisms would be of interest for future research.

Limitations of the study include whether the outcomes can be directly translated to humans at risk of developing atherosclerosis and whether the data obtained here can be generalized to the female gender. Furthermore, while the liver gene analyses indicated increased levels of *Saa*1 or *Tnf* was evident in mice with periodontitis it was not possible to detect these proteins in serum, which may be related to detection limits of the tests employed here. Therefore, more studies that further demonstrate casual linkages between severity of periodontitis and the initiation of atherogenesis are warranted.

In conclusion, the present study has demonstrated for the first time that periodontitis induces endothelial dysfunction in mice, which is consistent with the limited clinical studies performed to date in humans. The increased mRNA expression of *Tnf* and *Saa1* in liver tissues in response to the periodontal infection may provide a potential pathway between the extravascular periodontal inflammation and the systemic modulation of vascular function and energy metabolism. However, more direct data including the assessment of NOS activity and biochemical measurements of NO bioactivity are necessary to unambiguously demonstrate this proposed linkage.
